# Prohibitin 1 Regulates Inflammatory Mediators and Reactive Oxygen Species in Retinal Endothelial Cells

**DOI:** 10.3390/jcm11071915

**Published:** 2022-03-30

**Authors:** Li Liu, Youde Jiang, Jena J. Steinle

**Affiliations:** Department of Ophthalmology, Visual and Anatomical Sciences, Wayne State University School of Medicine, Detroit, MI 48201, USA; lliu@med.wayne.edu (L.L.); youdejiang55@gmail.com (Y.J.)

**Keywords:** endothelial cells, prohibitin 1, inflammation, retina

## Abstract

Diabetic retinopathy is associated with increased inflammatory mediator levels. In these studies, we focused on prohibitin 1. We performed western blotting for retinal lysates from diabetic mice and Epac1 floxed and cdh5Cre-Epac1 mice. We also grew primary retinal endothelial cells (REC) in normal (5 mM) and high (25 mM) glucose, and treated some cells with an Epac 1 agonist or prohibitin 1 siRNA. Western blotting was done to confirm knockdown of prohibitin 1 and Epac 1 agonism. We measured the tumor necrosis factor alpha (TNFα), interleukin-1-beta (IL-1β), phosphorylated prohibitin 1, phosphorylated nuclear factor kappa beta (NFkB), high mobility group box 1 (HMGB1) and reactive oxygen species (ROS) levels in REC after transfection with prohibitin 1 siRNA. Results showed that high glucose increased the inflammatory mediators, as well as HMGB1 and ROS. The levels of ROS, HMGB1, and inflammatory pathways were all reduced after cells were transfected with prohibitin 1 siRNA. Epac1 reduced prohibitin 1 phosphorylation. In conclusion, decreased prohibitin 1 significantly reduced the inflammatory mediator and ROS levels in REC. Epac1 regulates the prohibitin 1 levels in REC.

## 1. Introduction

There is an increasing appreciation that diabetic retinopathy is a result of activation or inhibition of a plethora of potential protein pathways. We chose to focus these studies on prohibitin 1 (PHB), as a study of a liver-specific prohibitin 1 knockout showed that prohibitin was protective against infection and inflammation [1]. Loss of prohibitin in the liver for 3 weeks led to severe injury, ROS, and apoptosis. Prohibitin 1 is a ubiquitously expressed and highly conserved protein, which is involved in mitochondrial actions, growth, and apoptosis [1]. Prohibitin 1 is primarily localized on the mitochondrial membrane [2]. Loss of prohibitin 1 on endothelial cells led to increased mitochondrial reactive oxygen species (ROS), which aided in angiogenic activities [2]. Similarly, loss of prohibitin 1 in a rat model of pulmonary hypertension showed that the loss of prohibitin 1 resulted in increased high mobility group box 1 (HMGB1)-mediated vascular injury [3]. In contrast to the liver and pulmonary systems, a study of the brain showed that inhibition of prohibitin 1 led to decreased neuroinflammation and interleukin-1-beta (IL1-β) levels [4]. The actions of prohibitin 1 are strongly regulated by post-translational modifications [5,6]. Prohibitin 1 has tyrosine kinase sites that can allow for phosphorylation, leading to increased signal transduction [6]. Prohibitin 1’s actions can also be regulated by O-GlyNAc medications and S-palmitoylation [5].

Less is known about prohibitin 1 in the eye. Prohibitin levels increased in the retina as a whole, but decreased in the retinal pigmented epithelial (RPE) cells of patients with age-related macular degeneration [7]. In that same paper, the authors reported that early diabetes led to decreased prohibitin 1 levels [7]. However, a more recent paper showed that the diabetic retina had decreased prohibitin 1 expression, but this paper did not investigate protein levels [8]. Another work has suggested that prohibitin 1 is regulated by aging and oxidative stress in the eye [9], suggesting that it may have complex actions in the eye. 

Another goal of the present study was to investigate whether the exchange protein for cAMP 1 (Epac1) regulates prohibitin 1. We have previously reported that Epac1 can reduce ROS, inflammatory pathways, and diabetes-induced damage to the retina [10,11,12]. Since Epac1 is protective to the retinal vasculature, we questioned whether Epac1 reduced prohibitin 1 levels in order to reduce the levels of ROS and inflammatory pathways in REC. There is little other literature on Epac1’s regulation of prohibitin 1. 

Based on the conflicting literature regarding prohibitin 1 in the eye and other organs, we wanted to determine whether prohibitin 1 regulates inflammatory proteins in primary human retinal endothelial cells (REC) grown in high glucose and in the retina of diabetic mice. 

## 2. Materials and Methods

*Diabetic Mice*. Male C57BL/6J mice were purchased from Jackson Laboratories (Bar Harbor, ME, USA) at 8 weeks of age. Mice were made diabetic by 60 mg/kg injections of streptozotocin (STZ) dissolved in citrate buffer for up to 5 consecutive days. Control mice received citrate buffer only. Glucose measurements were done biweekly, with glucose levels >250 mg/dL considered diabetic. Mice were not fasted before blood glucose measurements, and glucose measurements were taken on ~5 uL blood samples obtained from the tail vein, with samples measured by a hand-held measurement device. 

*Epac1 Endothelial Cell Specific Knockout Mice*. Epac1 floxed mice (B6; 129S2-Rapgef3^tm1Geno/J^ mice) and B6 FVB-Tg (cdh5-cre)7Mlia/J Cre mice were purchased from Jackson Laboratories. At 2 generations, Epac1 floxed mice were bred with cdh5-Cre mice, generating conditional knockout mice where Epac1 is eliminated in vascular endothelial cells [12]. At 2 months of age, male and female Epac1 floxed and Epac1 Cre-Lox mice were used to collect retinal samples. 

All animal procedures meet the Association for Research in Vision and Ophthalmology requirements, were approved by the Institutional Animal Care and Use Committee of Wayne State University, and conform to NIH guidelines.

*Retinal Endothelial Cells (REC)*. Primary human retinal endothelial cells (REC) were purchased from Cell Systems Corporation (CSC, Kirkland, Washington, DC, USA) and grown in Normal Glucose Cell Systems medium (5 mM) supplemented with microvascular growth factors (MVGS), 10 μg/mL gentamycin, and 0.25 μg/mL amphotericin B (Invitrogen, Carlsbad, CA, USA). Once cells reached confluence, some dishes were moved to the Cell Systems High Glucose Medium (25 mM glucose). Attachment factor was used for all cells, and only cells under passage 6 were used. Cells were quiesced by incubating in high or normal glucose medium without MVGS for 24 h before initiation of experiments. 

*Cell Treatments*. Some cells were treated with an Epac 1 agonist (8-CPT-2′-O-Me-cAMP, 10 μM, 24 h), as we have done in the past [13]. Other cells were transfected with prohibitin 1 siRNA (Qiagen, Hilden, Germany, [14]) or scrambled siRNA, as we have previously done [15]. 

*Western Blotting.* Whole retinal lysates from mice or cell culture lysates were collected into lysis buffer with protease and phosphatase inhibitors. Equal amounts of protein were separated onto a pre-cast tris-glycine gel (Invitrogen, Carlsbad, CA, USA) and blotted onto nitrocellulose membrane. After blocking in TBST (10 mM Tris-HCl buffer, pH 8.0, 150 mM NaCl, 0.1% Tween 20) and 5% (*w*/*v*) BSA, membranes were treated with Epac1, TNFα, IL-1β, HMGB1, and total prohibitin 1 (Abcam, Cambridge, MA, USA); phosphorylated (Ser536); and the total NFkB (Cell Signaling, Danvers, MA, USA), phosphorylated prohibitin 1 (Tyr 258, ThermoFisher, Waltham, MA, USA), or beta actin (Santa Cruz Biotechnology, Santa Cruz, CA, USA) antibodies followed via incubation with secondary antibodies labeled with horseradish peroxidase. Antigen–antibody complexes were visualized using a chemiluminescence reagent kit (Thermo Scientific, Pittsburgh, PA, USA). Data were acquired using an Azure C500 (Azure Biosystems, Dublin, CA, USA), and blot data were measured using Image Studio Lite software. 

*Reactive Oxygen Species (ROS) Assay.* ROS levels were measured using the fluorescent probe 2.7-dichloroflurescein diacetate (DCF-DA) (Invitrogen, Waltham, MA, USA). Briefly, cell lysates with 1 μg/uL proteinase inhibitor diluted in PBS were collected, and protein concentrations were calculated. Then, 10 μg protein samples were loaded in triplicate into a black 96 well plate. Next, 100 μL of proteinase inhibitor diluted in PBS and containing 5 μM fresh DCF-DA was added to the plate and incubated in 37 °C for 1 h. Fluorescence intensity was read on plate reader at excitation 485 nm and emission 530 nm. 

*ELISA Analyses for TNFα and IL-1β.* The TNFα ELISA (Life Technologies, Carlsbad, CA, USA) and IL-1β ELISA (R&D Systems, Menomomie, WI, USA) were done according to the manufacturer’s instructions, with the exception that both ELISAs were done overnight at 4C. 

*Statistics.* For cell culture work, a one-way ANOVA with Tukey’s post-hoc test was used. For the animal work, a *T*-test was used. For all data, a *p* < 0.05 was accepted as significant. Data were analyzed using Prism 8 (GraphPad, San Diego, CA, USA). 

## 3. Results

### 3.1. Prohibitin 1 Is Increased in the Diabetic Retina and in Retinal Lysates from cdh5Cre-Epac1 Mice

**Figure 1:**
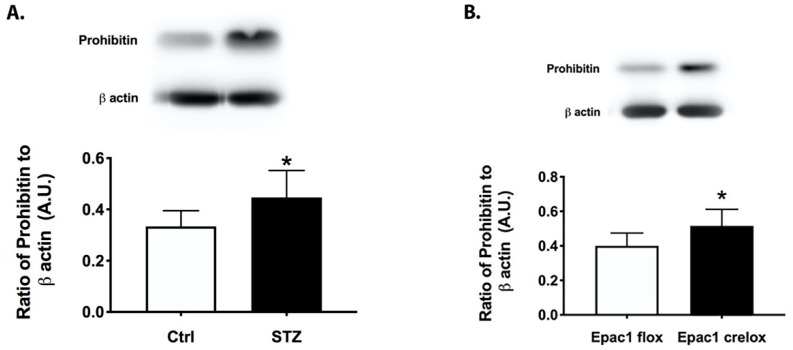
Prohibitin 1 is increased in the diabetic and Epac1 CreLox mouse retina. Western blot data on whole retinal lysates from control and STZ-treated (**A**) and Epac1 floxed and cdh5Cre-Epac1 floxed (**B**) mice. * *p* < 0.05 vs. control or Epac1 floxed. Data are mean ± SEM. N = 5.

We collected whole retinal lysates from two-month-old control and diabetic mice, as well as Epac1 floxed and cdh5-CrexEpac1 floxed mice. Figure 1A shows that the diabetic retina had significantly increased levels of prohibitin compared to those of the control mice. Figure 1B shows that loss of Epac1 increased prohibitin 1 levels. These data suggest that both diabetes and Epac1 may regulate total prohibitin 1 levels in the retina. 

### 3.2. An Epac1 Agonist Can Decrease Phosphorylation of Prohibitin 1 in REC Grown in High Glucose

**Figure 2:**
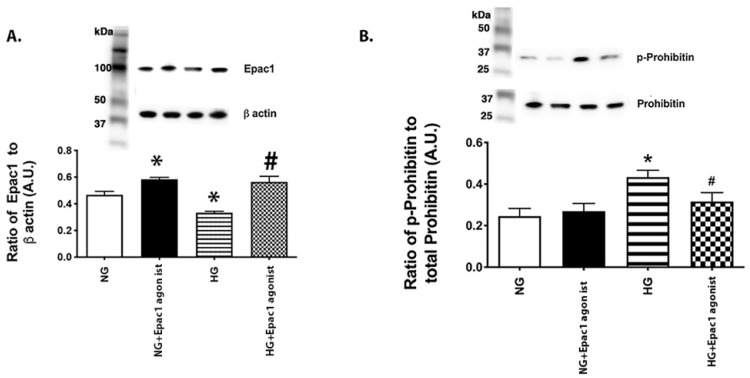
Epac1 agonist reduced phosphorylated prohibitin 1 levels in REC grown in high glucose. Western blot from retinal endothelial cells (REC) grown in normal (5 mM) or high (25 mM) glucose. Some cells in each condition were treated with an Epac1 agonist. Panel (**A**) shows successful increase in Epac1 with agonist, and Panel (**B**) shows the ratio of phosphorylated prohibitin 1 to prohibitin 1 levels. * *p* < 0.05 vs. NG, # *p* < 0.05 vs. HG. Data are mean ± SEM. N = 5.

To support our work in whole retinal lysates, we grew REC in normal (5 mM) and high (25 mM) glucose and treated some in each condition with an Epac1 agonist, as we have done in the past [16]. Figure 2A shows that the Epac1 agonist significantly increased Epac1 protein levels in cells grown in both the normal and high glucose. To investigate a potential mechanism by which Epac1 may regulate prohibitin 1 levels, we chose to measure the phosphorylation of prohibitin 1. Figure 2B shows that phosphorylated prohibitin 1 levels are significantly increased in high glucose, which was significantly reduced by treatment with the Epac1 agonist. 

### 3.3. Reduction in Prohibitin 1 Levels Inhibited Inflammatory Mediator Levels in REC Grow in High Glucose

**Figure 3:**
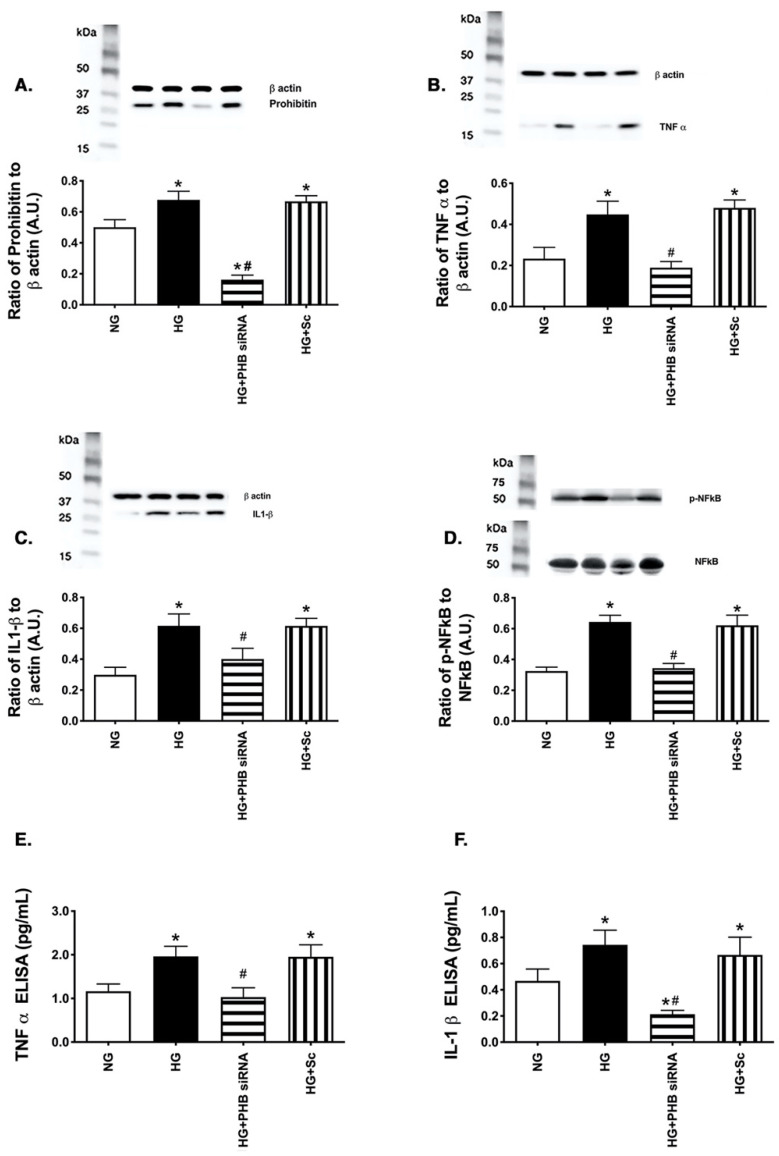
Reduced prohibitin 1 levels leads to diminished levels of TNFα, IL-1β, and phosphorylated NFkB. Western blot data from retinal endothelial cells (REC) grown in normal (5 mM) or high (25 mM) glucose. Some cells in each condition were treated with prohibitin 1 siRNA. Panel (**A**) is prohibitin 1, Panel (**B**) is TNFα, Panel (**C**) is IL-1β, and Panel (**D**) is phosphorylated to total NFkB protein levels. Panels (**E**,**F**) are ELISA results for TNFα (**E**) and IL-1β (**F**). * *p* < 0.05 vs. NG, *# p* < 0.05 vs. HG. N = 5. Data are mean ± SEM.

Since prohibitin 1 was increased in the diabetic retina, we wanted to investigate whether loss of prohibitin 1 could reduce inflammatory mediators. Figure 3A shows successful reduction in prohibitin 1 levels after siRNA transfection. Figure 3B–D shows that high glucose increased the TNFα, IL-1β and phosphorylation of NFkB, as expected. Figure 3E,F show ELISA results to support the western blot data for TNFα and IL-1β. In all cases, reduction of prohibitin 1 was protective to the cells, leading to diminished levels of key inflammatory mediators. 

### 3.4. Reduced Prohibitin 1 Decreased HMGB1 and ROS Levels in the REC Grown in High Glucose

**Figure 4:**
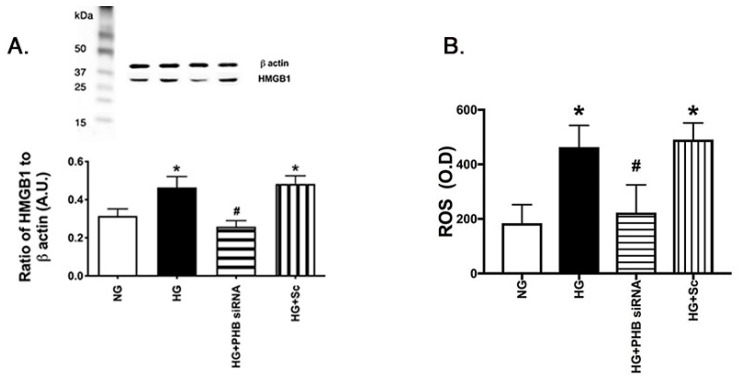
Prohibitin 1 siRNA reduced HMGB1 and ROS levels. Western blot data from retinal endothelial cells (REC) grown in normal (5 mM) or high (25 mM) glucose. Some cells in each condition were treated with prohibitin 1 siRNA. Panel (**A**) is HMGB1, Panel (**B**) is an ELISA for ROS. * p < 0.05 vs. NG, *# p* < 0.05 vs. HG. N = 5. Data are mean ± SEM.

We have previously reported that HMGB1 and ROS are increased in the diabetic retina and in REC grown in high glucose [10]. Figure 4 matches previous data showing that high glucose culturing conditions significantly increase HMGB1 and ROS. Both HMGB1 and ROS were decreased with REC when transfected with prohibitin 1 siRNA, suggesting that reduced prohibitin 1 levels reduce HMGB1 and ROS levels in the retinal vasculature. The data agree that prohibitin 1 regulates ROS in REC. 

## 4. Discussion

Our data from mice and primary retinal endothelial cell culture suggest that diabetes or high glucose conditions significantly increase prohibitin 1 protein levels. Our data show that high glucose increased prohibitin 1 phosphorylation in the REC, which was reduced by Epac1. We also found that reduction of prohibitin 1 levels by siRNA significantly decreased key inflammatory mediators. We also observed that reduced prohibitin 1 decreased high glucose-induced reactive oxygen species in the ROS assay. Our findings of increased prohibitin 1 levels in high glucose correspond to a study of murine mesangial glomerular cells, which found increased prohibitin 1 expression, as well as the acidic isoform of prohibitin 1 [17]. Reduced prohibitin 1 is also linked to increased HMGB1 and ROS levels in the pulmonary and hepatic systems [3]. A study of macrophages suggested that prohibitin 1 can increase inflammatory mediator levels through its actions on NFkB [18]. These findings all match our findings, which show that prohibitin 1 increases inflammatory mediator levels. 

In contrast to our work, one study found decreased prohibitin mRNA and protein levels in diabetic mice and rats, with increased prohibitin 1 protein levels in aging samples [8] and ARPE-19 epithelial cells [8], which is in contrast to our studies of endothelial cells. However, in contrast to this recent paper, others in the same group reported that aging reduced prohibitin 1 mRNA in 2010 [9]; thus it is unclear why the findings on prohibitin 1 have changed over time. In addition to studying the eye, studies of the heart and intestinal systems have demonstrated a protective effect of prohibitin 1 [19,20] with reduced TNFα levels in the intestinal epithelial cells [21]. 

Little has been done previously in the retinal vasculature, and nothing has been published investigating Epac1’s actions on prohibitin 1. It was unclear if Epac1 altered the prohibitin 1 levels for protective actions on retinal endothelial cells. We have previously shown that Epac1 is protective of retinal endothelial cells [10,11,12]. The literature suggests that prohibitin 1 actions are strongly regulated by post-translational modifications [5]. We found that Epac1 reduced phosphorylation of prohibitin 1 on Tyr 258 in REC in the present study. The data in this study suggest that Epac1 does reduce prohibitin 1 phosphorylation. Once reagents become more widely available, we will explore O-GlyNAc actions on this phosphorylation, as well as the downstream signaling from the tyrosine phosphorylation. 

Based upon the literature, prohibitin 1 actions may be cell specific or organ specific. A review study showed that adipocytes use prohibitin 1 primarily as a mitochondrial protein, while immune cells use prohibitin 1 for cellular signaling [22]. Our studies were done in endothelial cells vs. most other studies, which were completed in epithelial cells. Based on our findings and the literature, it is clear that additional work is needed to determine prohibitin 1’s primary mode of action in specific cell types. 

Since prohibitin 1 is embryonically lethal when eliminated [23], future goals include the generation of endothelial cell-specific knockout mice to determine the actions of prohibitin 1 in the retinal vasculature, followed by the induction of diabetes. We also appreciate that cdh5 Cre-Epac1 mice may have expression in blood or immune cells that confound our findings; however, our cell culture findings still support a role for Epac1’s regulation of prohibitin 1 in retinal endothelial cells. Future work will also further dissect whether ROS or inflammatory mediators are the key to prohibitin’s actions on the REC. This is the first report, that we are aware of, on prohibitin 1’s effects in REC. 

## 5. Conclusions 

In conclusion, our data suggest that prohibitin 1 is detrimental to the retinal vasculature. Reduction of prohibitin 1 led to decreased inflammatory mediators and ROS levels in REC. Epac1 regulated the phosphorylation of prohibitin 1 in primary REC. 

## Data Availability

All data is contained in the manuscript.
